# Comprehensive Genome‐Wide Analysis and Expression Profiling of *Pathogenesis‐Related Protein 1* (*PR‐1*) Genes in *Salvia miltiorrhiza*

**DOI:** 10.1002/fsn3.70117

**Published:** 2025-04-30

**Authors:** Huiyan Fan, Jingzhi Zhou, Qichao Wang, Minhui Zhang, Ziru Huang, Jiayi Han, Yiling Ying, Zhenming Yu, Guoyin Kai

**Affiliations:** ^1^ School of Pharmaceutical Sciences Zhejiang Chinese Medical University Hangzhou China; ^2^ College of Pharmaceutical Science Fujian University of Traditional Chinese Medicine Fuzhou China

**Keywords:** disease stress, gene family, hormone stress, *pathogenesis‐related 1*, *Salvia miltiorrhiza*

## Abstract

The *pathogenesis‐related 1 (PR‐1)* gene family is essential for plant defense and stress response. In this study, 11 *SmPR‐1* genes were identified in *Salvia miltiorrhiza* through comprehensive genomic analysis, all of which encoded proteins with conserved CAP (cysteine‐rich secretory protein, antigen 5, and pathogenesis‐related 1) domains and signal peptides. Phylogenetic analysis categorized these genes into five evolutionary clusters, reflecting their evolutionary divergence. Chromosomal localization analysis revealed that the *SmPR‐1* gene family is distributed across three chromosomes: Chr1 contains six genes, Chr6 contains three, and Chr8 contains one. Intraspecific collinearity analysis indicated segmental duplications of *SmPR‐1‐5* and *SmPR‐1‐11* on Chr1. Interspecific collinearity analysis showed that five *SmPR‐1* genes are collinear with both *Arabidopsis thaliana* and *Scutellaria baicalensis*, with *SmPR‐1‐1* also exhibiting collinearity with *Oryza sativa* and *Zea mays* . Tissue‐specific expression profiling indicated high expression levels in the flowers and stems, indicating their roles in various developmental processes. Differential expression patterns under hormonal and biotic stress revealed that *SmPR‐1‐5* was particularly responsive to brassinosteroid (BR) treatment. Subcellular localization analysis indicated that SmPR‐1‐5 was present in both the cytoplasm and nucleus, suggesting its involvement in intracellular signaling. Additionally, CMV infection triggered a time‐dependent expression pattern, activating specific genes during the early and late infection stages. These findings provide valuable insights into the functional roles of *SmPR‐1* genes in stress responses and immunity, laying the groundwork for breeding disease‐resistant *S. miltiorrhiza* varieties. Future research should explore the regulatory mechanisms and interactions of *SmPR‐1* genes with other defense pathways to fully understand their contribution to plant resistance.

## Graphical abstract

This study identified 11 SmPR‐1 genes in *Salvia miltiorrhiza* , which play crucial roles in plant defense and stress response. Expression profiling and subcellular localization of SmPR‐1‐5 suggest its involvement in intracellular signaling, and CMV infection triggers specific gene activation patterns. The findings pave the way for developing disease‐resistant *S. miltiorrhiza* varieties by understanding the functional roles of SmPR‐1 genes.

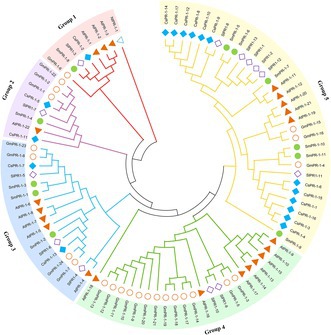

## 1 Introduction

The plant immune response is a multifaceted system designed to mitigate the harmful effects of pathogenic microorganisms and prevent infections and tissue damage. It consists of two primary defense strategies: pattern recognition‐based immunity (PRBI), which is the first line of defense, and effector‐triggered immunity (ETI), which presents a more specific and robust response. Additionally, pathogenesis‐related (PR) genes that encode PR proteins act synergistically to enhance plant resistance to pathogen invasion. The synergistic interplay between these mechanisms enables plants to mount durable and robust immune responses (Silva et al. 2018; Takken and Tameling 2009; Wan et al. 2019). Pathogenesis‐related (PR) genes represent a distinct group of genetic elements activated in plants during pathogen encounters and exposure to abiotic stressors. These genes play vital roles in plant defense by initiating protective cascades and encoding PR proteins that are essential for disease resistance (Hamamouch et al. 2010; Punja 2001; Sung et al. 2021). These genes are not only expressed during microbial infections but also contribute to critical plant processes, including maturation, flowering, plasmolysis, and senescence (Breen et al. 2017; Zribi et al. 2021). PR proteins enhance plant tolerance to pathogens by accumulating various peptides and proteins (Punja 2001). Moreover, they can respond to diverse abiotic stressors, including drought, freezing, ultraviolet radiation, salinity, osmotic stress, and hormonal signals such as salicylic acid (SA), abscisic acid (ABA), jasmonic acid (JA), and auxin (IAA) (Agrawal et al. 2000; Akbudak et al. 2020; AlHudaib et al. 2022; Ghorbel et al. 2021; Mou et al. 2003). Characterized by their small molecular size, heat stability, and protease resistance, PR proteins are broadly expressed in diverse plant tissues (van Loon et al. 1994). First discovered in the 1970s in tobacco plants infected by the tobacco mosaic virus (TMV) and induced by salicylic or acetylsalicylic acid (Van Loon and Van Kammen 1970), the PR protein family has since expanded into 17 distinct groups based on similarities in amino acid sequences, immunological relationships, and catalytic functions (Sels et al. 2008).

The PR‐1 protein family is the first identified pathogenesis‐related protein and is widely distributed in monocotyledonous and dicotyledonous plants (Akbudak et al. 2020; Fraser 1981; Kothari et al. 2016; Lawrence et al. 1996; Li et al. 2023; Shin et al. 2014; Zhang et al. 2022a). Renowned for its pivotal role in systemic acquired resistance (SAR), PR‐1 marks the plant's defense state by facilitating programmed cell death (Breen et al. 2017; Chassot et al. 2007). Highly conserved across plant species, PR‐1 proteins are classified into acidic and basic types based on their isoelectric point (Breen et al. 2017). These proteins perform dual functions: they are either secreted into the extracellular space or stored in the vacuoles (Sessa et al. 1995). A defining feature of PR‐1 proteins is the conserved CAP (cysteine‐rich secretory protein, antigen 5, and pathogenesis‐related 1) domain, comprising approximately 150 amino acid residues that form a stable structure of four α‐helices and four β‐strands reinforced by disulfide bonds (Breen et al. 2017). The CAP domain imparts antimicrobial activity by recognizing and binding to sterols on pathogen membranes and inhibiting their growth (Chen et al. 2014; Schneiter and Di Pietro 2013). This structural element is essential for PR‐1 the physiological roles in countering biological and environmental stresses (Ghorbel et al. 2021). Accumulation of PR‐1 proteins in plant tissues is strongly associated with pathogen infection. For instance, in TMV‐infected tobacco leaves, PR‐1 proteins account for 1%–2% of the total leaf protein (Alexander et al. 1993). In tomatoes, PR‐1c suppresses spore germination and germ tube growth of *Phytophthora infestans* , reducing disease incidence (Niderman et al. 1995). These findings demonstrate that PR‐1 proteins are critical intermediaries in plant defense. In tea plants, PR‐1 genes respond actively to stress from blister blight disease, linking closely with key signaling pathways, including the TCA cycle, NPR1‐mediated signaling, EDS16, BGL2, PR4, and HCHIB (Zhang et al. 2022a).

PR‐1 genes are essential for plant immunity against diseases and play a significant role in abiotic stress management. In wheat, the TaPR‐1‐1 gene can be induced by cold, salt, and osmotic stress, with its expression enhancing stress tolerance in yeast and Arabidopsis (Wang et al. 2019). In tomatoes, PR‐1b1 is upregulated under low temperatures, and its overexpression in transgenic plants improves stress adaptation (Goyal et al. 2016; Kiba et al. 2007; Sarowar et al. 2005). In rice, OsSAP1 protein activates endogenous stress‐related genes, including OsAMTR1, SCP/TAPS, and the pathogenesis‐related protein OsSCP (Kothari et al. 2016). In *Arabidopsis*, the drought‐induced transcriptional regulator Di19 increases the expression of pathogenesis‐related genes, further reinforcing stress resilience (Liu et al. 2013). The transcription factor NTL6, a member of the NAC family, directly binds to the promoter regions of cold‐responsive pathogenesis‐related genes, such as PR‐1, PR‐2, and PR‐5, stimulating their expression (Seo et al. 2010). These PR genes are regulated by abiotic stresses as well as by plant hormones and chemical inducers, including ET, SA, JA, brassinosteroids (BR), benzothiadiazole (BTH), and isonicotinic acid (INA) (Breen et al. 2017; Chandrashekar et al. 2018; Edreva and Kostoff 2005; Van Loon and Van Strien 1999; Zhang et al. 2015). In summary, PR‐1 genes play a crucial role in plant response to diverse biotic and abiotic stresses, and their transcription is tightly controlled by various regulatory factors.

*Salvia miltiorrhiza* is a perennial herb of the genus *Salvia* in the Lamiaceae family that serves as a valuable component of traditional Chinese medicine, and its roots are commonly used for medicinal purposes (Sui 2019). Renowned for treating cardiovascular diseases, it promotes blood circulation, regulates menstruation, calms the mind, and alleviates swelling and pain (Wu and Wang 2012). Its short lifespan, robust growth, advanced genetic modification techniques, compact genome, and small chromosome number render *S. miltiorrhiza* an ideal model for pharmaceutical plant research (Boli 2009; Xu et al. 2016; Zhang et al. 2015). Extensively cultivated in China, its expanding planting area has been increasingly threatened by diseases, including fungal infections, which affect yield and quality (Lu et al. 2021; Wang et al. 2018; Zhang et al. 2022b). Despite the crucial role of the PR‐1 gene family in plant disease resistance, its specific functions in *S. miltiorrhiza* remain insufficiently explored. In this study, we characterized the SmPR‐1 gene family using genomic data from *S. miltiorrhiza* and conducted an in‐depth bioinformatic analysis. Through transcriptome analysis and quantitative real‐time PCR (qRT‐PCR), we examined the expression profiles of SmPR‐1 genes in cucumber mosaic virus infection and plant hormone treatment to identify genes with significant functional roles. This study provides a theoretical foundation for further studies on the biological functions of the *SmPR‐1* gene family and suggests novel strategies for disease management and quality improvement in this medicinal plant.

## 2 Results

### 2.1 Identification of Members of the *SmPR‐1* Gene Family

In the *S. miltiorrhiza* genome, 11 *SmPR‐1* sequences were identified using the NCBI Batch CD‐Search tool and designated as *SmPR‐1‐1* through *SmPR‐1‐11*. These sequences contained the CAP domain, which was characteristic of cysteine‐rich secretory proteins, component 5 antigens, and PR‐1 proteins. The sequence lengths varied, with SmPR‐1‐8 being the longest at 210 amino acids, whereas SmPR‐1‐5, SmPR‐1‐9, and SmPR‐1‐10 were the shortest at 159 amino acids. The majority (90.91%) of the sequences ranged from 150 to 200 amino acids. The molecular weights (MWs) of these proteins ranged from 17.44 to 23.74 kDa, and their theoretical isoelectric points (PIs) varied from 4.56 to 9.8 (Table 1). All SmPR‐1 proteins were identified as hydrophilic, as indicated by their negative GRAVY indices, and signal peptides were detected for each sequence. Sequence alignment revealed a high similarity between SmPR‐1 proteins. Subcellular localization predictions suggested that the SmPR‐1 proteins were predominantly located in the chloroplast and extracellular spaces, which were consistent with observations in other plant species (Table S1).

**TABLE 1:** Protein sequence characterization and information on physicochemical properties of *SmPR‐1*.

Sequence ID	Gene	Protein length (aa)	MW (kDa)	pI	GRAVY	*E*‐value HMM search	Subcellular location		SP	CDS length	
SMILT018280.1			SmPR‐1‐1	165	18.30	9.64	−0.278	6.30E‐22	Chloroplast	26–27	498
SMILT018281.1			SmPR‐1‐2	188	20.46	6.72	−0.08	2.00E‐19	Chloroplast	24–25	567
SMILT008035.1			SmPR‐1‐3	167	18.62	8.97	−0.18	1.20E‐20	Chloroplast	25–26	504
SMILT009945.1			SmPR‐1‐4	170	18.52	8.38	−0.252	5.10E‐16	Chloroplast	22–23	513
SMILT013753.1			SmPR‐1‐5	159	17.68	8.27	−0.29	6.60E‐20	Extracell	22–23	480
SMILT013754.1			SmPR‐1‐6	161	18.12	9.04	−0.466	2.30E‐20	Extracell	24–25	486
SMILT013755.1			SmPR‐1‐7	161	17.44	6.64	−0.207	7.70E‐24	Extracell	23–24	486
SMILT014473.1			SmPR‐1‐8	210	23.74	9.8	−0.358	1.60E‐26	Chloroplast	26–27	633
SMILT015244.1			SmPR‐1‐9	159	17.76	5.51	−0.377	1.30E‐21	Extracell	21–22	480
SMILT015245.1			SmPR‐1‐10	159	17.76	4.56	−0.393	1.70E‐20	Chloroplast	24–25	480
SMILT015247.1			SmPR‐1‐11	164	18.38	5.16	−0.336	9.00E‐23	Extracell	20–21	495

### 2.2 Comparative Sequence Alignment and Phylogenetic Analysis of SmPR‐1 Proteins

**FIGURE 1:**
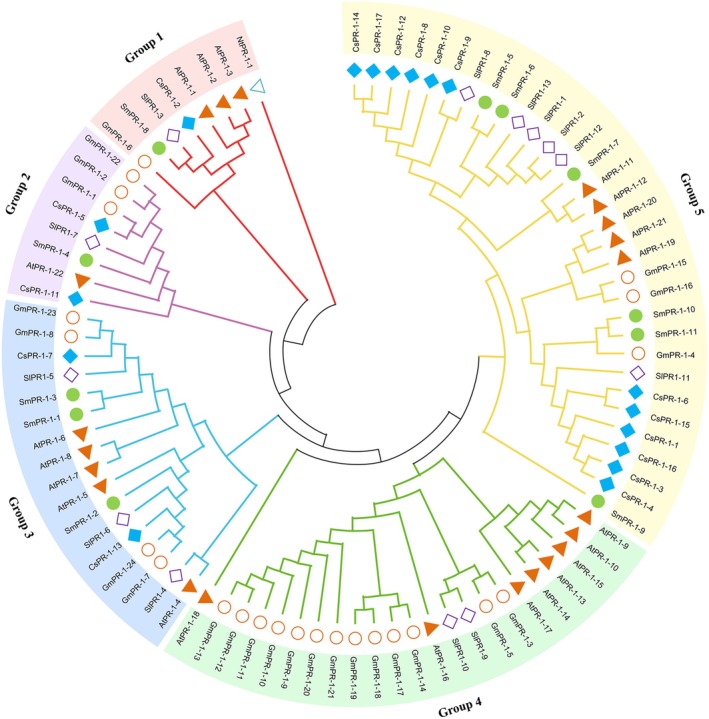
Phylogenetic tree of the PR‐1 gene family from *Salvia miltiorrhiza* , *Arabidopsis thaliana* , *Camellia sinensis* , *Solanum lycopersicum* , *Glycine max* , and *Nicotiana tabacum* . The phylogenetic tree was constructed using the Neighbor‐Joining (NJ) method in MEGA version 11, with 1000 bootstrap replicates to evaluate branch support. Each species is represented by a unique combination of color and shape: *S. miltiorrhiza* genes are indicated by green circles, *A. thaliana* genes by red triangles, *C. sinensis* genes by blue squares, *S. lycopersicum* genes by purple‐bordered squares, *G. max* genes by orange‐bordered circles, and *N. tabacum* genes by blue‐bordered triangles.

**FIGURE 2:**
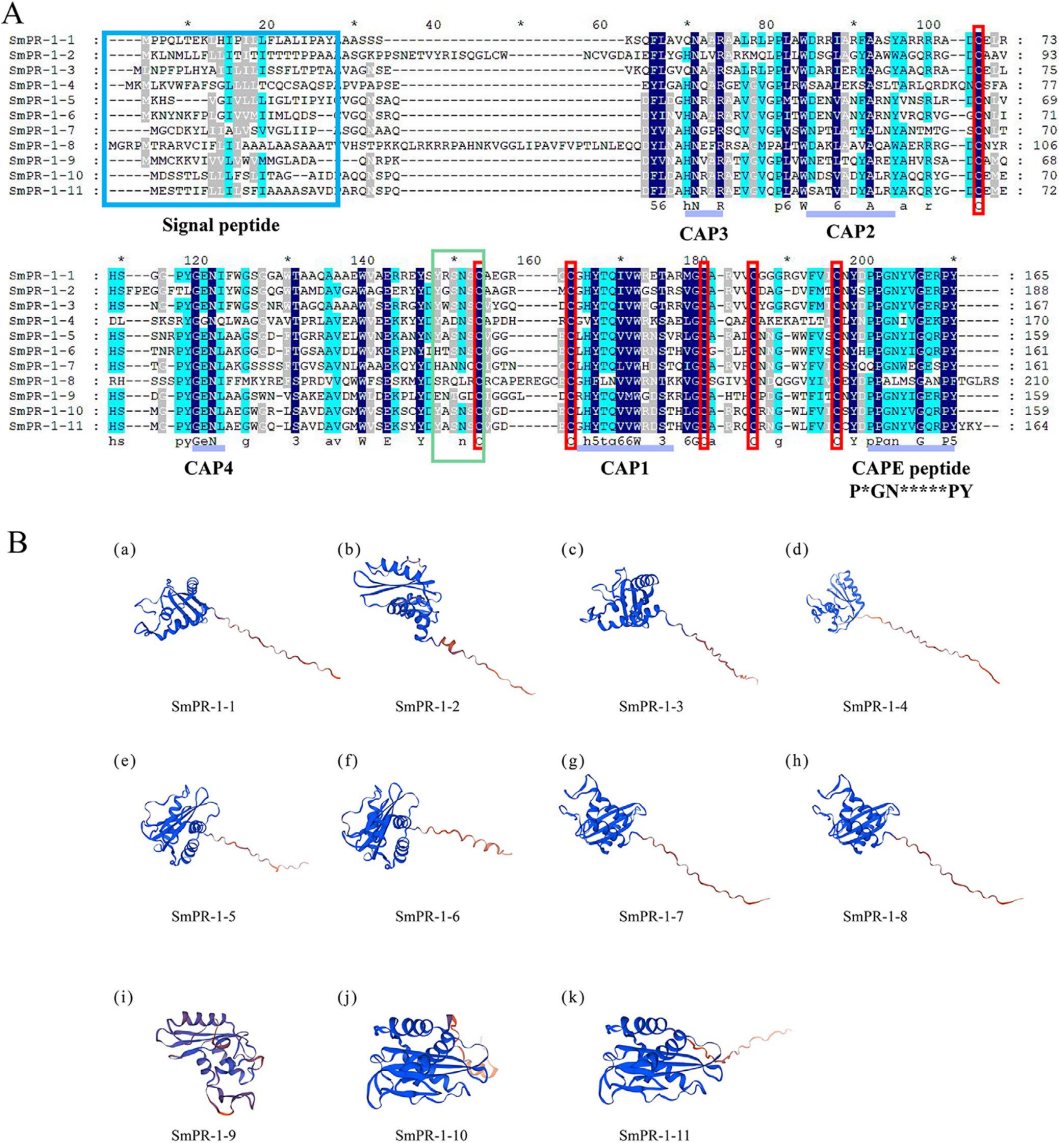
Multiple sequence alignment and predicted tertiary structures of GmPR‐1 proteins. (A) Multiple sequence alignment of SmPR‐1s. The purple line represents CAP‐derived peptide (CAPE). The blue rectangle was the signal peptide sequence which was distinguished in the SignalP 4.1 server. The green rectangle represented the caveolin‐binding motif (CBM). The red rectangle represents six cysteine motifs. (B) Predicted 3D structure of the SmPR‐1 protein. Blue represents N‐terminus and red represents C‐terminus.

Phylogenetic analysis of PR‐1 genes from *Salvia miltiorrhiza* (Sm), *Arabidopsis thaliana* (At), *Camellia sinensis* (Cs), *Solanum lycopersicum* (Sl), *Glycine max* (Gm), and *Nicotiana tabacum* (Nt) revealed significant evolutionary relationships and functional divergence within this gene family. Among the 88 PR‐1 sequences analyzed, five distinct groups emerged, representing unique evolutionary lineages. Groups 1 and 2 contained a single *SmPR‐1* gene (*SmPR‐1*, *SmPR‐8*, and *SmPR‐4*), highlighting their relatively isolated evolutionary status. Group 3, encompassing three *SmPR‐1* genes (*SmPR‐1‐1*, *SmPR‐2*, and *SmPR‐3*), suggested potential functional redundancy or specialization among closely related members. Group 5 was the largest group, including six *SmPR‐1* genes (*SmPR‐1‐5*, *SmPR‐6*, *SmPR‐7*, *SmPR‐9*, *SmPR‐10*, and *SmPR‐11*), possibly indicating recent duplication events and subsequent diversification (Figure 1). Sequence alignment revealed that most SmPR‐1 proteins possess six conserved cysteine residues that are crucial for structural stability and pathogen resistance. However, incomplete CAP‐derived peptides (CAPEs) in SmPR‐1‐4 and SmPR‐1‐8 suggested that unique evolutionary modifications potentially affected pathogenic interactions. Additionally, Groups 3 and 5 formed distinct phylogenetic clusters characterized by high intragroup similarity and divergent evolutionary trajectories (Figure 2A), potentially reflecting functional specialization or adaptation to specific environmental challenges. The prediction of the three‐dimensional structures of 11 SmPR‐1 proteins revealed that proteins from *SmPR‐1* genes that are phylogenetically close tend to have more similar structures, particularly those within the same group. For example, *SmPR‐1‐10* and *SmPR‐1‐11* in Group 5 exhibited high structural similarity (Figure 2B), which is consistent with the results from motif analysis and gene structure analysis.

### 2.3 Chromosome Localization and Collinearity Analysis of *SmPR‐1*

**FIGURE 3:**
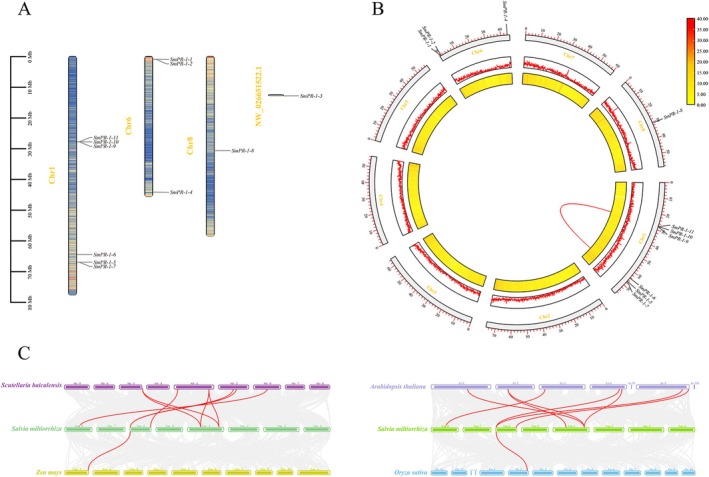
Chromosomal localization and collinearity analysis of SmPR‐1 genes. (A) Chromosomal localization map. The chromosomal length scale is shown on the left side, with chromosome names displayed in yellow font, and SmPR‐1 genes labeled in black font. (B) Intraspecific collinearity analysis of SmPR‐1 genes. Collinear genes are connected by red lines, and the color scale represents the distribution of SmPR‐1 genes. (C) Interspecific collinearity analysis of SmPR‐1 genes. Collinear genes are connected by red lines.

Chromosomal localization analysis revealed that among the eight chromosomes, only three contained *SmPR‐1* genes: six on Chr1, three on Chr6, and one on Chr8, with one unassembled sequence (Figure 3A). Intraspecific collinearity analysis showed that among the 11 *SmPR‐1* genes, only *SmPR‐1‐5* and *SmPR‐1‐11* exhibited segmental duplications, both of which were located on Chr1(Figure 3B). Synonymous and nonsynonymous mutation ratio (Ka/Ks) analysis of the duplicated segments revealed a Ka/Ks value of less than 1, suggesting that these genes might have undergone purifying selection. Interspecific collinearity analysis of the *SmPR‐1* gene family with *A. thaliana* , *Scutellaria baicalensis*, *Oryza sativa* , and *Zea mays* revealed that the *SmPR‐1* genes family is collinear with all four species. Further analysis showed that the highest number of collinear gene pairs occurred with *A. thaliana* and *S. baicalensis* (eight pairs each), while the lowest was observed with *O. sativa* and *Z. mays* (one pair each). Notably, five *SmPR‐1* genes (S*mPR‐1‐1*, *SmPR‐1‐4*, *SmPR‐1‐5*, *SmPR‐1‐8*, and *SmPR‐1‐11*) exhibited interspecific collinearity with both *A. thaliana* and *S. baicalensis* , and one *SmPR‐1* gene (*SmPR‐1‐1*) showed interspecific collinearity with all four species (Figure 3C).

### 2.4 Analysis of Structural and Conserved Motifs of *SmPR‐1*

**FIGURE 4:**
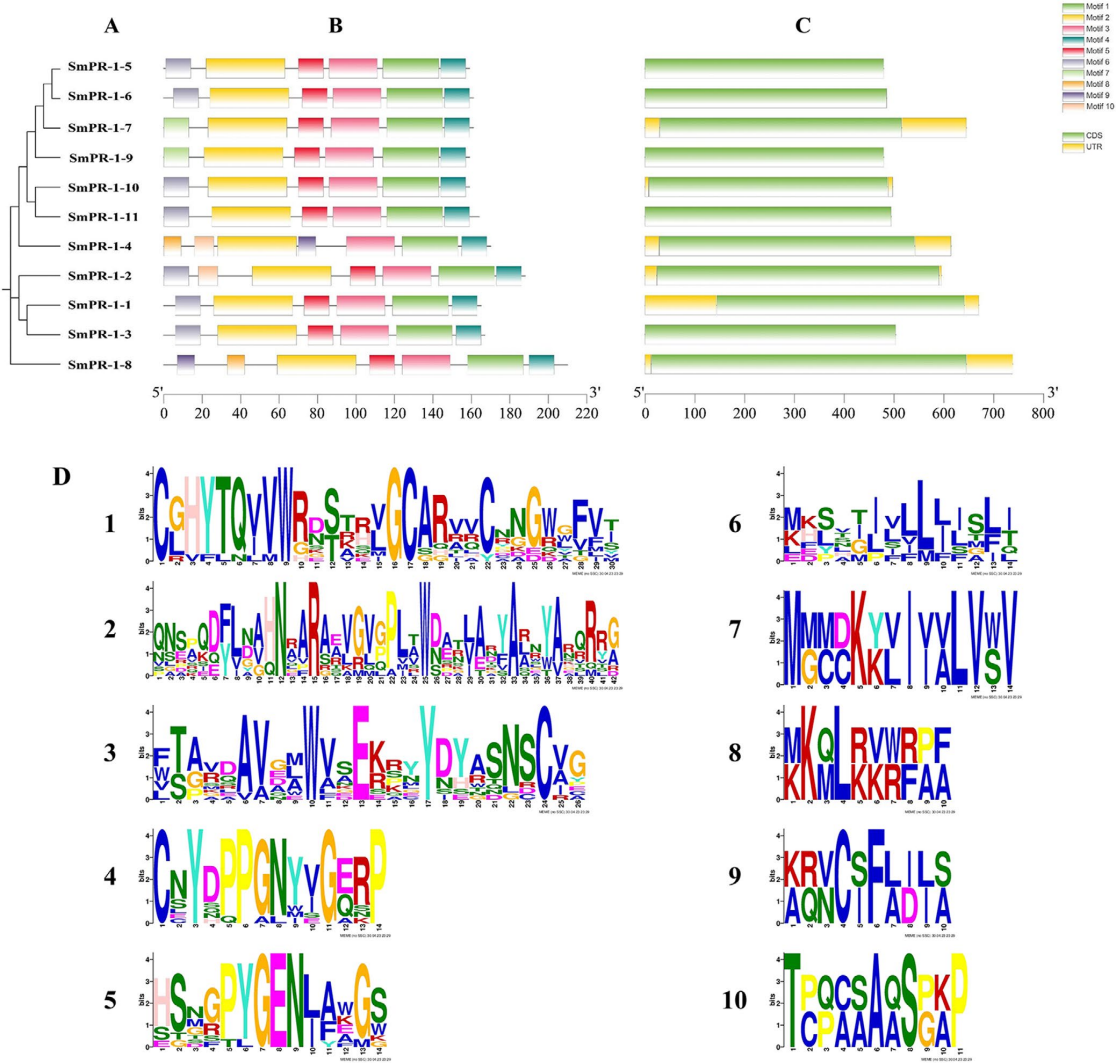
Phylogenetic tree, extron–intron structure, and conserved motif identification of the *SmPR‐1* genes. (A) The phylogenetic tree includes 11 sequences. (B) Ten different conserved motifs are represented by different colors. (C) Exon–intron structure of the SmPR‐1 gene family. The yellow boxes represent noncoding sequences, and the green boxes indicate introns. (D) The motif sequences of the *SmPR‐1* genes.

Structural and motif analyses of *SmPR‐1* genes were conducted using TBtools software to gain deeper insights into their characteristics. Motif prediction results identified motifs 1, 2, 3, and 4 in all the genes, which were consistently arranged, indicating high conservation and their likely role as CAP domains. Motifs 5 and 6 were also relatively conserved and appeared in most of the sequences. Unique motifs were identified: motif 7 was exclusive to SmPR‐1‐4 and SmPR‐1‐8, motif 10 to SmPR‐1‐2 and SmPR‐1‐4, and motif 9 to SmPR‐1‐4 and SmPR‐1‐8. DNA sequence analysis revealed that all SmPR‐1 genes contained a single exon of a similar length, with no introns present in any gene (Figure 4).

To further understand the SmPR‐1 gene family in *S. miltiorrhiza* , we conducted detailed analyses of the gene structure and motif composition using TBtools software. The analysis identified 10 distinct motifs (motifs 1–10) within the *SmPR‐1* gene family. Notably, motifs 1, 2, 3, and 4 were consistently present across all *SmPR‐1* genes in a uniform arrangement, indicating a high degree of conservation and suggesting that their role as the core components of the conserved CAP domains is essential for PR‐1 protein function (Figure 3). Motifs 5 and 6 were identified as relatively conserved and were present in the majority of *SmPR‐1* sequences, supporting the hypothesis of functional conservation within this gene family (Figure 3C). In contrast, certain motifs exhibited restricted distribution, indicating potential functional divergence. For instance, motif 7 was unique to *SmPR‐1‐4* and *SmPR‐1‐8*; motif 10 appeared only in *SmPR‐1‐2* and *SmPR‐1‐4*; and motif 9 was exclusive to *SmPR‐1‐4* and *SmPR‐1‐8* (Figure 4B,D). These unique motif patterns may indicate specialized roles in responding to specific environmental cues or developmental processes. Structural analysis revealed that all *SmPR‐1* genes possessed a single exon with no introns, and exon lengths were relatively uniform across the family (Figure 4C). This conserved single‐exon structure was aligned with other PR‐1 genes in other plant species, demonstrating their evolutionary conservation and potential regulatory efficiency.

### 2.5 Prediction of *Cis*‐Acting Elements and Transcription Factors Among the *SmPR‐1* Genes

**FIGURE 5:**
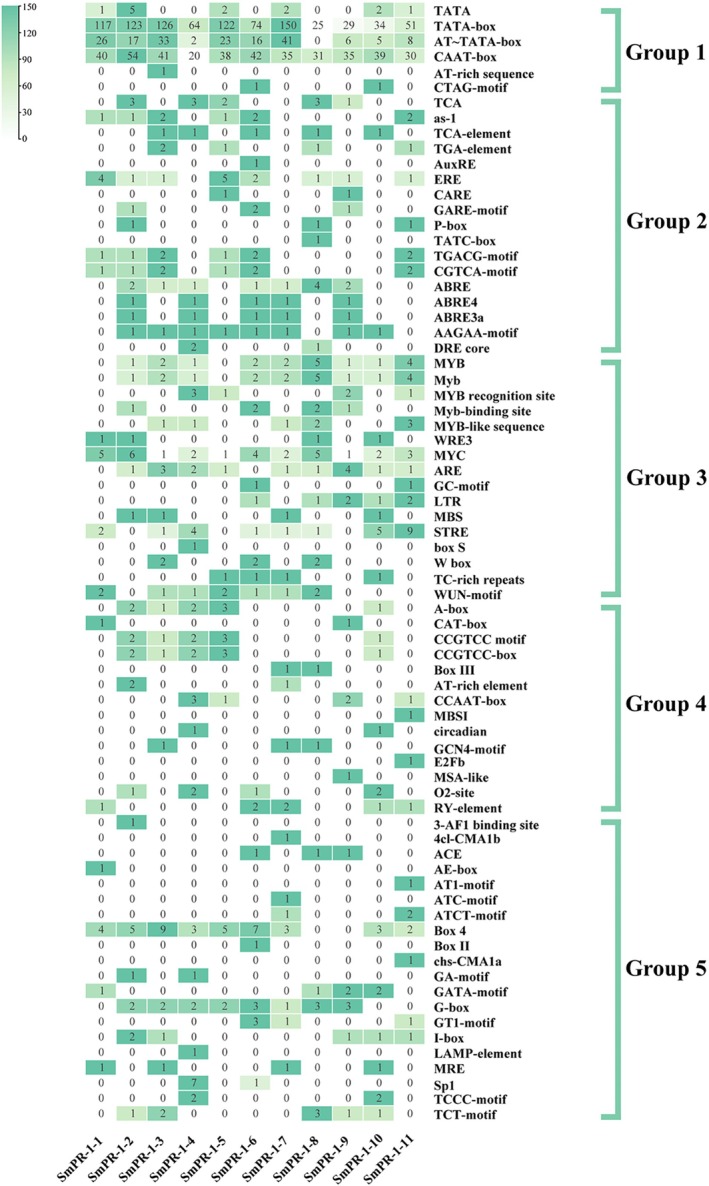
Cis‐regulatory elements of the *SmPR‐1* genes family. Each row represents a different type of CRE. The number in each box indicates the quantity of each CRE. A 0‐to‐1 scale method was adopted to better illustrate the variation in CRE quantities. The blank indicates the absence of the corresponding element. We categorized all the CREs into five groups based on their main functions.

Analysis of the *cis*‐regulatory elements (CREs) within the promoter sequences of *SmPR‐1* genes identified a diverse array of CREs, categorized into five functional classes: general transcription‐related elements (Group 1), hormone‐responsive elements (Group 2), stress‐responsive elements (Group 3), growth and development‐related elements (Group 4), and light‐responsive elements (Group 5). Multiple hormone‐responsive elements were detected, including those responsive to SA (TCA, as‐1, and TCA element), IAA (AuxRE and TGA element), ET (ERE), gibberellin (GA) (CARE, GARE motif, p‐box, and TATC box), methyl jasmonate (MeJA) (TGACG motif and CGTCA motif), and ABA (ABRE, ABRE4, ABRE3a, AAGAA motif, and DRE core) (Figure 5). SA‐ and MeJA‐related CREs, strongly linked to defense mechanisms, were particularly abundant in most *SmPR‐1* genes. Additionally, stress‐responsive elements, such as MYB‐binding sites and W boxes, were prominent, indicating the potential involvement of *SmPR‐1* genes in both biotic and abiotic stress responses.

### 2.6 Transcriptional Profiling of *SmPR‐1* Genes Across Various Tissues

**FIGURE 6:**
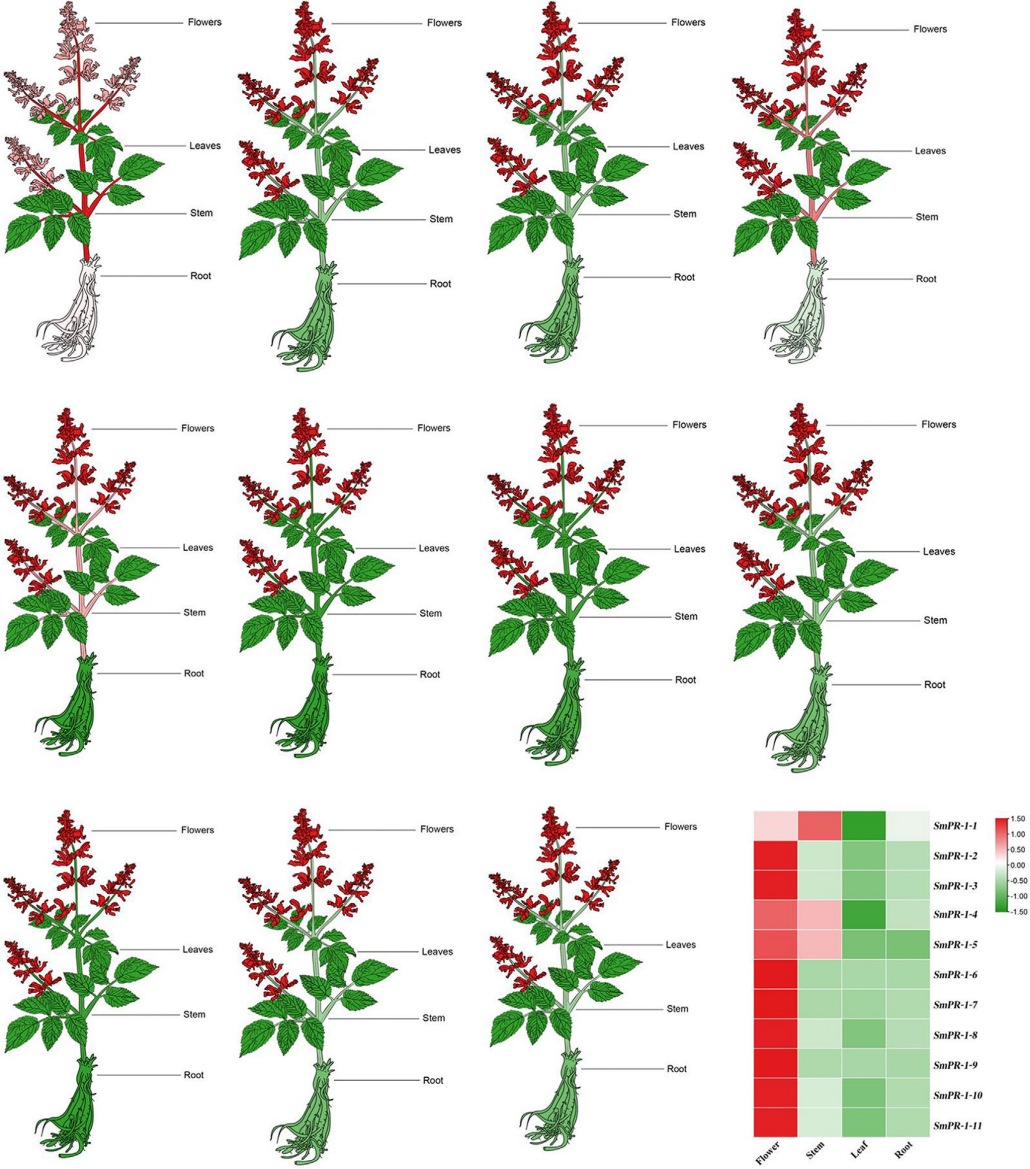
Expression profiles of the *SmPR‐1* genes family across various tissues in *S miltiorrhiza*. The diagram illustrates the distinct organs of *S. miltiorrhiza* , including roots, stems, leaves, and flowers. Heatmaps were generated using TBtools, with the heatmap on the right depicting expression levels, where red indicates high expression and green indicates low expression.

The expression patterns of *SmPR‐1* genes in *S. miltiorrhiza* revealed distinct organ‐specific characteristics, suggesting potential functional divergence. Among the 11 *SmPR‐1* genes, *SmPR‐1‐1*, *SmPR‐1‐4*, and *SmPR‐1‐9* exhibited particularly high expression levels in the flowers, indicating their possible roles in flower development and reproductive processes. Additionally, *SmPR‐1‐1* and *SmPR‐1‐9* were significantly expressed in the stems, suggesting their involvement in specific biological processes within this tissue. Most *SmPR‐1* genes showed low expression levels in leaves and roots, with the exception of *SmPR‐1‐1*, which displayed moderate expression in the roots (Figure 6). These findings indicate the diverse functions of *SmPR‐1* genes, emphasizing their organ‐specific expression in the flowers, stems, and roots.

### 2.7 Transcriptional Response of *SmPR‐1* Genes to CMV Infection

**FIGURE 7:**
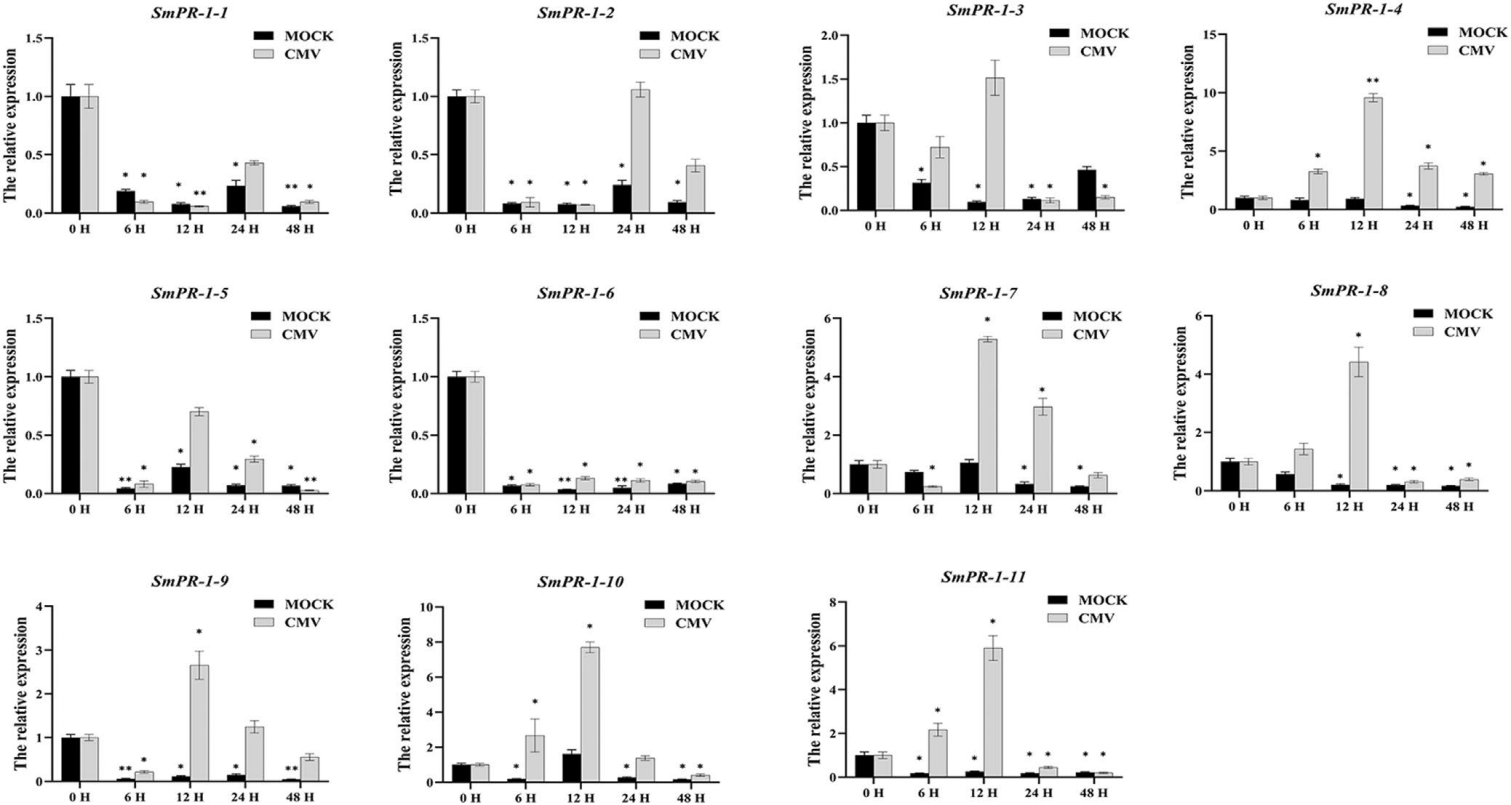
qRT‐PCR analysis of SmPR‐1 gene expression in response to CMV infection at five time points (0, 6, 12, 24, and 48 h). The mean expression values were calculated from three independent biological replicates and are normalized to mock‐inoculated controls.

qRT‐PCR analysis evaluated the transcriptional profiles of *SmPR‐1* genes in *S. miltiorrhiza* following *cucumber mosaic virus* (CMV) infection at five time points: 0, 6, 12, 24, and 48 h postinfection. These results revealed distinct expression patterns across the *SmPR‐1* gene family. Notably, *SmPR‐1‐4*, *SmPR‐1‐7*, and *SmPR‐1‐9* demonstrated a significant increase in expression at 12 and 24 h postinfection, with elevated levels persisting at 48 h (Figure 7), suggesting their involvement in sustained defense responses against CMV. Conversely, *SmPR‐1‐10* and *SmPR‐1‐11* exhibited an early and rapid expression response, peaking at 6 and 12 h, followed by a decline at 24 h, indicating their role in the initial defense against viral infection. These variations highlight a complex, multiphase defense mechanism against CMV in *S. miltiorrhiza* .

### 2.8 Hormonal Stress‐Induced Expression of *SmPR‐1* Genes

**FIGURE 8:**
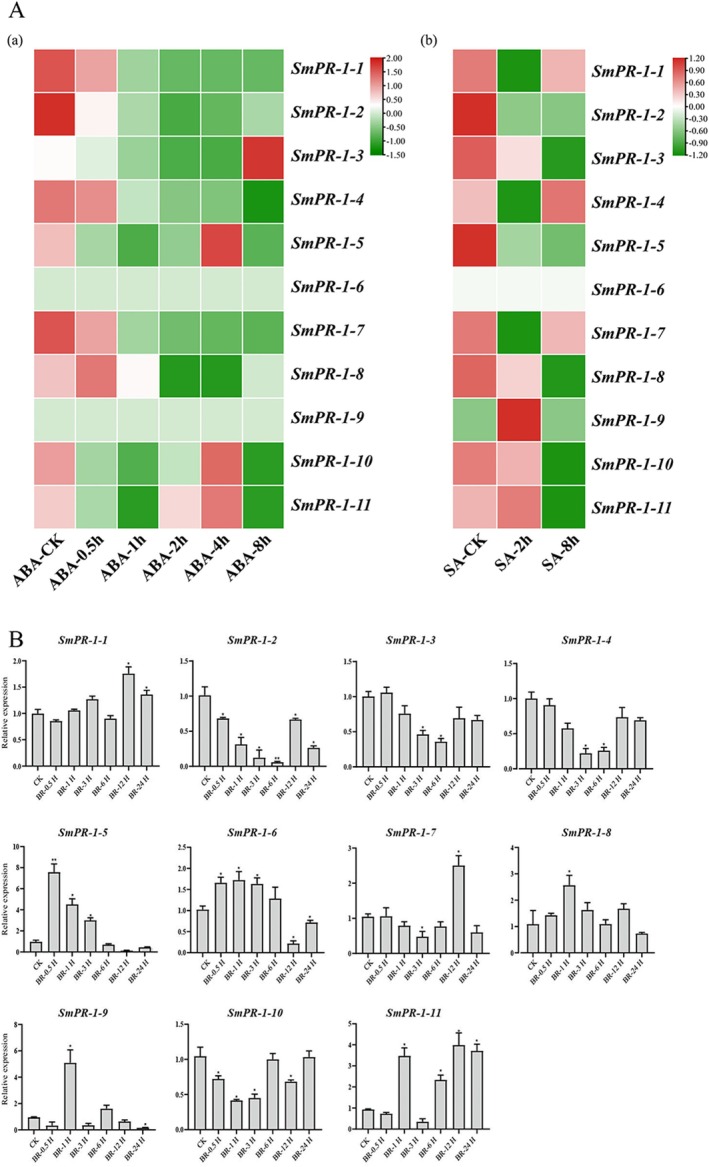
Comparison of the transcript expression results from RNA‐seq and qRT‐PCR analysis of the *SmPR‐1* genes induced by exogenously applied phytohormones (ABA, SA, and BR). A. Expression of *SmPR‐1* genes in response to ABA treatment was measured at six time points (0, 0.5, 1, 2, 4, and 8 h), while expression in response to SA treatment was assessed at three time points (0, 2, and 8 h). B. qRT‐PCR analysis of *SmPR‐1* gene expression in response to BR treatment, monitored at seven time points (0, 0.5, 1, 3, 6, 12, and 24 h).

Identification of *cis*‐regulatory motifs in the *SmPR‐1* promoter region has revealed numerous hormone‐responsive elements, highlighting the potential involvement of *SmPR‐1* genes in hormone‐mediated signaling pathways. Transcriptome analysis of *S. miltiorrhiza* further confirmed the responsiveness of specific *SmPR‐1* genes to abscisic acid (ABA) and SA treatments, demonstrating their role in complex hormonal regulation. The *SmPR‐1* gene family exhibits distinct time‐dependent responses to these hormones. Under the influence of ABA treatment, the *SmPR‐1‐3* gene exhibits a significant upregulation trend after 8 h of treatment, while the *SmPR‐1‐5* gene shows an increase in expression after 2 h of treatment, peaking at 4 h. In contrast, the expression levels of *SmPR‐1‐1*, *SmPR‐1‐4*, and *SmPR‐1‐7* genes gradually decrease after treatment, indicating their likely involvement in ABA signaling pathways and related biological processes. Under the treatment of SA, the expression levels of *SmPR‐1‐1*, *SmPR‐1‐4*, and *SmPR‐1‐7* genes initially increased after 2 h of SA treatment but then showed a declining trend after 8 h. In contrast, the expression levels of *SmPR‐1‐2*, *SmPR‐1‐3*, *SmPR‐1‐5*, and *SmPR‐1‐10* genes continuously decreased (Figure 8A). These findings reveal the complex and dynamic expression changes of the *SmPR‐1* gene family under SA induction, highlighting their potential roles in plant defense responses.

The transcriptional responses of *SmPR‐1* genes to BR hormone treatment revealed significant temporal and gene‐specific variations. *SmPR‐1‐5*, *SmPR‐1‐8*, and *SmPR‐1‐9* exhibited rapid induction at early time points (0.5 and 1 h), with *SmPR‐1‐5* displaying the highest expression level at 0.5 h, followed by a gradual decline. In contrast, *SmPR‐1‐1* and *SmPR‐1‐7* exhibited moderate increases in expression at mid‐term time points (6 h), which decreased at later stages. Notably, *SmPR‐1‐2* and *SmPR‐1‐4* showed reduced expression at early time points but gradually increased expression at later stages (Figure 8B). These results demonstrated the specific and differential responses of *SmPR‐1* genes to BR treatment, suggesting distinct functional roles in BR‐mediated biological processes.

### 2.9 Subcellular Localization Analysis of SmPR‐1 Protein

**FIGURE 9:**
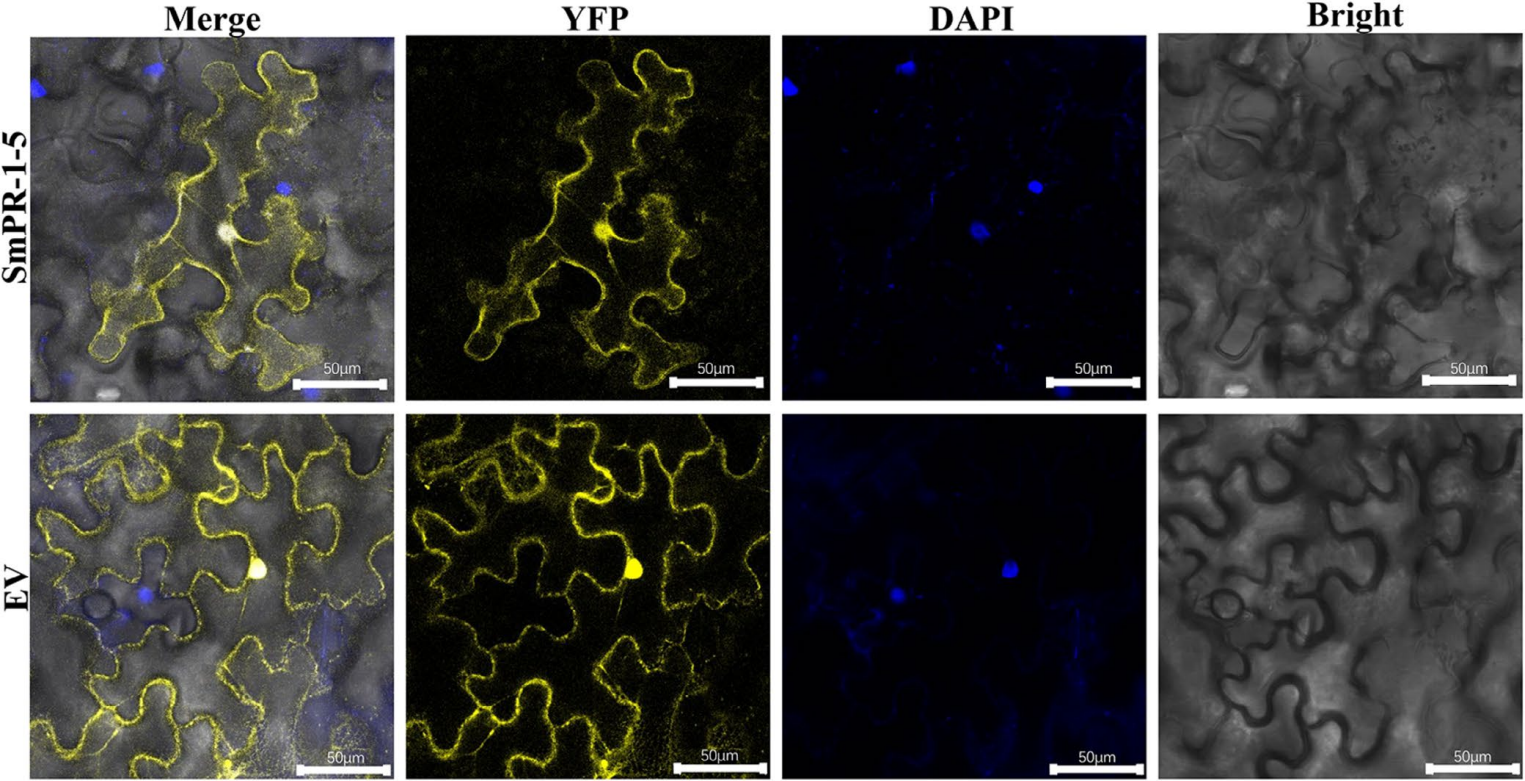
Subcellular localization of SmPR‐1‐5 protein in *Nicotiana benthamiana* lower epidermal cells. Images of epidermal cells were captured using visible light, blue fluorescence, yellow fluorescence, and merged light. *Agrobacterium rhizogenes* carried the recombinant vector pHB‐SmPR‐1‐5‐YFP and the empty vector pHB‐YFP with the yellow fluorescent protein (YFP) tag. DAPI was used to label the nucleus.

Bioinformatics analysis of SmPR‐1 amino acid sequences predicted that most SmPR‐1 proteins are localized in either the chloroplast or extracellular space. Among the SmPR‐1 gene family, SmPR‐1‐5 showed the strongest expression in response to BR stress, as determined by qRT‐PCR, making it a candidate for detailed subcellular localization studies using laser confocal microscopy. DAPI was used as a nuclear stain, and pHB‐YFP served as a fluorescent marker. Remarkably, the results revealed that SmPR‐1‐5 was localized not only to the cell membrane but also within the nucleus (Figure 9), exceeding initial expectations. This dual localization suggests a complex functional role of SmPR‐1‐5, which integrates extracellular defense with intracellular regulatory functions. These findings provide new insights into the versatility of PR‐1 family proteins and highlight SmPR‐1‐5 as a key mediator of both defense signaling and gene regulation in *S. miltiorrhiza* .

## 3 Discussion

PR‐1 proteins are among the most abundantly synthesized proteins during plant immune responses, serve as key indicators of heightened defense signaling, and play a vital role in systemic acquired resistance (SAR) (van Loon et al. 2006; Van Loon and Van Strien 1999). Rapid advancements in comprehensive genomic sequencing methodologies have prompted investigations of the PR‐1 gene family across a wide range of plant species (Akbudak et al. 2020; AlHudaib et al. 2022; Chu et al. 2022; van Loon et al. 2006; Lu et al. 2011; Yin et al. 2023; Zhang et al. 2022a; Zribi et al. 2023). Research has highlighted the pivotal role of the *PR‐1* gene family in plant defense mechanisms against diseases. *Salvia miltiorrhiza* is a perennial herb widely utilized in traditional medicine and is susceptible to numerous pests and diseases. Reliance on chemical pesticides for pest control risks fostering pest resistance and leaves residues that degrade the quality of medicinal materials, thereby affecting therapeutic efficacy (Wang et al. 2018). Consequently, identifying resistance genes within the *S. miltiorrhiza* genome and developing disease‐resistant varieties are essential for enhancing its medicinal value and clinical efficacy. Despite its importance, molecular studies on PR‐1 genes in *S. miltiorrhiza* remain unexplored. This study represents the first comprehensive genomic analysis of *PR‐1* genes in *S. miltiorrhiza* with the aim of elucidating their roles in biotic and abiotic stress responses.

This study identified 11 PR‐1 proteins in the genome of *S. miltiorrhiza* , a relatively low number compared to that in other dicotyledonous and monocotyledonous plants. This discrepancy may result from functional redundancy among PR‐1 members, suggesting that genome size is not directly correlated with the number of PR‐1 genes (AlHudaib et al. 2022). Phylogenetic analysis of the PR‐1 gene family across seven species ( *Arabidopsis thaliana* , *Camellia sinensis* , *Solanum lycopersicum* , *Glycine max* , *Nicotiana tabacum* , *Triticum aestivum* , and *S. miltiorrhiza* ) classified the 111 PR‐1 genes into five clades. The 11 SmPR‐1 genes were distributed across four clades with conserved motifs, reflecting evolutionary adaptation to specific environmental pressures. Notably, *S. miltiorrhiza* had the highest number of PR‐1 genes in clade 5 (Figure 2), suggesting a critical role for this group in defense mechanisms. The classification of PR‐1 genes in *S. miltiorrhiza* showed both shared and unique features compared with other species. For instance, the 23 OsPR‐1 genes in rice have been grouped into acidic (Group II) and basic (Group III) proteins based on their isoelectric points (pI), with Group II containing acidic proteins and Group III containing basic proteins (Liu and Xue 2006). Similarly, 23 TaPR‐1 genes in wheat were classified into acidic, basic, and basic proteins with C‐terminal extensions (Lu et al. 2011), while tea PR‐1 genes were categorized into five clades based on signal peptides, pI, and C‐terminal extensions (Zhang et al. 2022a). In contrast, the *S. miltiorrhiza* PR‐1 genes encoding acidic and basic proteins clustered within the same group, similar to the findings of Akbudak et al. (2020) and sugarcane (Chu et al. 2022), where PR‐1 proteins clustered regardless of their acidic or basic nature.

PR‐1 proteins are generally classified into two groups based on their isoelectric points (pIs): acidic and basic (Kiraga et al. 2007). Among the 11 PR‐1 proteins identified in *S. miltiorrhiza* , nine were basic and two acidic. Basic PR‐1 proteins are typically associated with strong antimicrobial activity. For instance, in tomatoes, the basic PR‐1c and PR‐1g proteins can exhibit greater antimycotic activity than the acidic PR‐1a and PR‐1b proteins (Niderman et al. 1995). Similarly, overexpression of the basic *PR‐1* gene from pepper in tobacco significantly enhances resistance to heavy metal stress and pathogenic bacteria (Sarowar et al. 2005). In tea, 10 of the 17 CsPR‐1 proteins are basic and rapidly expressed during blister blight infection. Although the acidic *SmPR‐1‐10* and *SmPR‐1‐11* genes showed an early response to infection, their expression declined significantly during later stages (Figure 6). These varied expression patterns suggest a complex defense mechanism against CMV. Notably, all the 11 *SmPR‐1* genes identified in this study were intronless (Figures 1 and 3), which may facilitate rapid transcriptional regulation under stress. The absence of introns is hypothesized to be an evolutionary adaptation that enables quicker responses to environmental pressures, as observed in tea, where the intronless *CsPR‐1* gene is expressed more rapidly during Fusarium wilt stress (Zhang et al. 2022a). Additionally, *SmPR‐1‐6*, *SmPR‐1‐7*, *SmPR‐1‐8*, and *SmPR‐1‐9* lack a caveolin‐binding motif (CBM), potentially impairing their ability to bind sterols and affecting their antimicrobial activity.

Analysis of the promoter regions of *SmPR‐1* genes revealed numerous *cis*‐regulatory elements associated with stress responses, developmental stages, and hormonal signaling pathways (Figure 4). Notably, MYB and MYC transcription factor‐binding sites, which regulate plant growth, development, metabolism, and responses to biotic and abiotic stressors, are prevalent (Cao et al. 2020; Wang et al. 2024). The abundance of these elements suggests that these genes are likely to be involved in diverse molecular and metabolic pathways. As indicator genes for systemic acquired resistance (SAR), the *PR‐1* family is essential for plant defense against biological stress (Ali et al. 2018), and their accumulation enhances resistance to adverse conditions (Zribi and Brini 2020). In this study, *SmPR‐1‐4*, *SmPR‐1‐7*, and *SmPR‐1‐9* exhibited significantly increased expression at 12 and 24 h post‐CMV infection, maintaining high levels during later stages. In contrast, *SmPR‐1‐10* and *SmPR‐1‐11* were rapidly expressed at 6 h, peaked at 12 h, and declined by 24 h. These distinct expression patterns indicate varied roles of PR‐1 genes in the antiviral mechanisms of *S. miltiorrhiza* , aligning with the findings in tea (Zhang et al. 2022a) and Tibetan barley(Yin et al. 2023). Elevated *PR‐1* expression has been shown to enhance the resistance of *A. thaliana* to *Peronospora parasitica* and wheat to rust infections (Niki et al. 1998; Zhang et al. 2015). Additionally, genes such as *TaLr35PR1*, *ZmPR‐1*, and *ScPR1* modulate defensive responses in wheat, corn, and sugarcane against foliar rust (Li et al. 2016), *Magnaporthe oryzae* (Shi 2020), and *Acidovorax avenae* (Chu et al. 2022), respectively.

The plant immune system relies on two primary signaling pathways, JA and SA, which are essential for disease resistance through synergistic or antagonistic interactions (Glazebrook 2005; Zhang et al. 2010). Phytohormones can activate the transcription of *PR‐1* genes, which are pivotal for plant defense (Pieterse et al. 2009). In *A. thaliana* , PR‐1 gene expression is regulated by SA or INA (2,6‐dichloroisonicotinic acid) but not by MeJA or ET (Durrant and Dong 2004). Conversely, PR‐1 expression in tobacco requires a combination of SA, ET, and MeJA, because neither MeJA nor ET alone is sufficient (Xu et al. 1994). In rice, PR‐1b is weakly induced by SA but strongly induced by JA (Mei et al. 2006), whereas the *BjPR1* gene in mustard responds significantly to SA but not to JA or ABA (Ali et al. 2018). In potatoes, PR‐1‐5 is induced by *Phytophthora* infection, ABA, and IAA (Zaynab et al. 2021). In maize, *ZmPR‐1* is strongly induced by SA, MeJA, and ABA (Ma et al. 2022). Our research revealed that the *SmPR‐1* gene family in *S. miltiorrhiza* contained ABA and SA response elements and exhibited varied responses to ABA, SA, and BR, suggesting a potential synergistic role in plant resistance mechanisms.

Extensive research has demonstrated that *PR‐1* genes respond to both biotic and abiotic stresses, underscoring their essential roles in managing environmental challenges (Liu et al. 2013; Mitsuhara et al. 2008; Seo et al. 2008; Zeier et al. 2004). Additionally, evidence suggests that PR‐1 proteins function beyond stress responses and contribute to plant growth and development (Breen et al. 2017; Fraser 1981; Hanfrey et al. 1996). Their presence in senescent leaves of mature flowering plants and their accumulation in the sepals of developing flowers indicate their key roles in the plant life cycle (Lotan et al. 1989). In maize, 17 *ZmPR‐1* genes are differentially expressed across various biological processes, highlighting the functional diversity of the PR‐1 gene family in plant physiology (Ma et al. 2022). In our study, *SmPR‐1* genes displayed significantly higher expression in the flowers than in the leaves of *S. miltiorrhiza* (Figure 5), suggesting their critical roles in flower development and immune defense in this species.

## 4 Materials and Methods

### 4.1 Identification and Characterization of PR‐1 Genes

The genome sequence of *S. miltiorrhiza* was sourced from a publicly available database (Ma et al. 2021). To identify members of the *PR‐1* gene family, we used the Pfam database (http://pfam.xfam.org/, accessed on 26 November 2023) for analysis (Mistry et al. 2020; Sonnhammer et al. 1997) to download the HMM profile (PF00188), which represents the conserved domain specific to this gene family. A homology search was performed using the HMMER tool with an *e*‐value threshold of 1e^−10^, followed by a BLASTP search to consolidate potential PR‐1 sequences. The presence of CAP domains in the identified sequences was verified using the NCBI CDD database (https://www.ncbi.nlm.nih.gov/cdd/, accessed on 27 November 2023) (Marchler‐Bauer et al. 2016) and the Pfam database, and sequences lacking completely conserved domains were excluded. SignalP 5.0 (https://services.healthtech.dtu.dk/service.php?SignalP‐5.0, accessed on 27 November 2023) was employed to predict the N‐terminal signal peptides and their cleavage sites (Armenteros et al. 2019). Additional gene characteristics, including isoelectric point (pI), molecular weight (MW), grand average hydropathy (GRAVY), and coding sequence length (CDS), were analyzed using the ExPASy ProtParam tool (https://web.expasy.org/protparam/, accessed on 28 November 2023) (Table 1) (Gasteiger 2005). Subcellular localization of SmPR‐1 proteins was predicted using WoLF PSORT (https://wolfpsort.hgc.jp/, accessed on 28 November 2023) (Armenteros et al. 2019).

### 4.2 Comparative Sequence Alignment and Evolutionary Analysis

To investigate the evolutionary relationships among PR‐1 proteins, the phylogenetic analysis was conducted using 88 PR‐1 proteins from six species: *S. miltiorrhiza* (11 SmPR‐1 proteins), *Solanum lycopersicum* (13 SlPR‐1 proteins), *Camellia sinensis* (17 CsPR‐1 proteins), *Glycine max* (24 GmPR‐1 proteins), *Arabidopsis thaliana* (22 AtPR‐1 proteins), and *Nicotiana tabacum* (1 NtPR‐1 protein). Multiple sequence alignment was performed using ClustalW with default settings, followed by neighbor‐joining (NJ) phylogenetic analysis conducted in MEGA 11 (v11.0.13) software to construct the phylogenetic tree (Tamura et al. 2021). The robustness of the tree topology was validated using bootstrap analysis with 1000 replications.

### 4.3 Chromosomal Distribution and Synteny Analysis of the *SmPR‐1* Gene Family

The distribution of *SmPR‐1* genes across chromosomes was examined using genome annotation data, with unassembled scaffolds removed from the genome files. The selective pressure on gene duplication events was assessed by calculating the Ka/Ks ratio using TBtools software. To explore the patterns of gene duplication among *SmPR‐1* transcription factors, collinearity analysis was conducted using MCScanX and visualized with TBtools. For the synteny analysis, genomic data from multiple species were employed, including *Oryza sativa* (rice): PRJDB1747, https://asia.ensembl.org/index.html; *Zea mays* (maize): PRJEB32225, https://www.ncbi.nlm.nih.gov/; and *Scutellaria baicalensis* (baikal skullcap): GWHBJEC00000000, https://ngdc.cncb.ac.cn/?lang=zh.

### 4.4 Analysis of Gene Structure, Stable Domain Composition, and Protein 3D Structure Prediction

The architectural features of the SmPR‐1 gene family were analyzed using the Gene Structure Display Server 2.0 (GSDS) available at http://gsds.gao‐lab.org/ (accessed on 30 November 2023). Conserved motifs were identified using the MEME tool configured to detect up to 10 motifs with widths between 30 and 70 amino acids, while all other parameters were kept at their default settings (https://meme‐suite.org/meme/, accessed on 30 November 2023). The protein 3D structure prediction of the SmPR‐1 gene family was performed using the SWISS‐MODEL platform (https://swissmodel.expasy.org/, accessed on 23 January 2025), with default settings.

### 4.5 Promoter *Cis*‐Acting Elements and Transcription Factors Prediction

To identify *cis*‐regulatory elements (CREs) and transcriptional regulators of the *SmPR‐1* genes, 2000 bp sequences upstream of the transcription start site for each gene were extracted from the genomic data. The PlantCARE database (http://bioinformatics.psb.ugent.be/webtools/plantcare/html/, accessed on 04 December 2023) was used to analyze the promoter regions of CREs (Lescot 2002).

### 4.6 Hormonal Treatments and CMV Inoculation

The leaves of 1‐month‐old *S. miltiorrhiza* seedlings were harvested, and the margins and petioles were trimmed and inoculated with *Agrobacterium rhizogenes* (C58C1 strain) to induce hairy root growth. The resulting hairy roots were transferred to conical flasks containing 100 mL of half‐strength MS nutrient solution and incubated on a shaker at 25°C and 100 rpm. After approximately 40 days, when sufficient hairy root biomass was achieved, the cultures were treated with 50 μM·L^−1^ BR solution. Samples were collected at six time intervals: 0.5, 1, 3, 6, 12, and 24 h posttreatment. Immediately after collection, the samples were flash‐frozen in liquid nitrogen (−196°C) and stored at −80°C for RNA isolation. For CMV infection, the infectious cDNA clones were transformed into *Agrobacterium tumefaciens* strain GV3101 and infiltrated into newly expanded leaves of *S. miltiorrhiza* via agroinfiltration at an OD_600_ of 1.0 (Zhou et al. 2021). Leaves from CMV‐infected and mock‐inoculated plants were harvested 2 weeks postinoculation. All experiments were performed in triplicate to ensure biological replicates.

### 4.7 RNA Isolation and cDNA Preparation

Total RNA was extracted from the samples using a milling method and purified using an RNA Easy Fast Plant Tissue Total RNA Isolation Kit (TIANGEN, China). The purified RNA was subjected to reverse transcription using the Evo M‐MLV RT Mix Kit (Accurate Biology, China) to synthesize complementary DNA (cDNA). The resulting cDNA was stored at −80°C for subsequent analyses.

### 4.8 RNA‐Seq and qRT‐PCR Detection

Transcriptome data for *S. miltiorrhiza* tissues were obtained from our laboratory (currently unpublished), and additional transcriptome data for samples treated with ABA, MeJA, and SA were obtained from the BioProject database using the identifiers PRJNA703309 (ABA), PRJNA393563 (MeJA), and PRJNA301529 (SA) (https://www.ncbi.nlm.nih.gov/, accessed on 16 December 2024). To construct the regulatory map of the *SmPR‐1* family, the protein sequences of the *SmPR‐1* family were used as query sequences, and the BLAST function in TBtools (v2.154) was employed to search for matching sequences of *SmPR‐1* family proteins in transcriptome protein libraries. The FPKM values of the matching sequences were then extracted. To present the regulatory trends more intuitively, the FPKM values for different time points of the same treatment were normalized using a normal distribution function (Z = (X—μ)/σ, where *X* is the FPKM value, *μ* is the mean, and *σ* is the standard deviation), and the expression heatmap was visualized using TBtools (v2.154) (Chen et al. 2020). The primer sequences for the *SmPR‐1* genes were designed using Real‐time PCR (TaqMan) and Probes Design Tool (https://www.genscript.com/tools/real‐time‐pcr‐taqman‐primer‐design‐tool, accessed on 13 December 2023) (Table S1). The expression levels of *SmPR‐1* genes were quantified via qRT‐PCR using cDNA synthesized from flower tissue as the template and *actin* as the internal control. Each reaction contained 1 μL of template cDNA, 0.5 μL each of upstream and downstream primers, 5 μL of 2X SYBR Green Pro Taq HS Premix (Accurate Biology, China), and 3 μL of ddH_2_O for a total reaction volume of 10 μL. The qRT‐PCR protocol included an initial denaturation at 95°C for 30 s, followed by 40 cycles of 5 s at 95°C and 30 s at 60°C, with fluorescence monitoring. Melt curve analysis was performed with steps at 95°C for 15 s, 60°C for 1 min, and 95°C for 1 s, with fluorescence recorded at each step. Gene expression was quantified by the 2^‐ΔΔCt^ method. Each sample was measured in triplicate to ensure the reliability of the results.

### 4.9 Gene Cloning and Subcellular Localization Analysis

The coding sequence (CDS) of *SmPR‐1‐5* was cloned into the pHB transient expression vector (Table S1), which contained a yellow fluorescent marker. Agroinfiltration assays were conducted according to standard protocols (Shi et al. 2022). Leaves from 1‐month‐old *N. benthamiana* plants were selected for infiltration, and the bacterial suspensions were inoculated into the lower leaf epidermis and marked accordingly. After infiltration, plants were maintained under optimal conditions, including a 24‐h dark incubation at 25°C, followed by a 24‐h light incubation. An empty vector prepared under identical conditions served as a control. The subcellular localization of *SmPR‐1‐5* proteins was examined using laser confocal fluorescence microscopy, and green fluorescence emission was recorded using a Zeiss LSM 880 laser scanning confocal microscope. To confirm these results, the WoLF PSORT online tool (http://wolfpsort.seq.cbrc.jp/, accessed on 05 March 2024) was used to predict the subcellular localization of *SmPR‐1* encoded proteins.

## 5 Conclusions

This study conducted a comprehensive genome‐wide investigation of the *PR‐1* gene family in *S. miltiorrhiza* and identified 11 *SmPR‐1* genes through bioinformatic analyses. These genes encode proteins with conserved CAP domains and signal peptides. Phylogenetic analysis grouped the *SmPR‐1* genes into five distinct clades, indicating evolutionary divergence and potential functional specialization. Chromosomal localization analysis shows that the *SmPR‐1* gene family is distributed on Chr1 (6 genes), Chr6 (3 genes), and Chr8 (1 gene). Intraspecific collinearity analysis identifies segmental duplications of *SmPR‐1‐5* and *SmPR‐1‐11* on Chr1. Interspecific analysis indicates that five *SmPR‐1* genes are collinear with *A. thaliana* and *S. baicalensis* . Tissue‐specific expression profiling revealed high expression levels of certain *SmPR‐1* genes in flowers and stems, suggesting their role in developmental processes. Subcellular localization studies demonstrated that *SmPR‐1‐5* is present in both the cytoplasm and nucleus, implying its involvement in intracellular signaling pathways. Expression analyses under biotic and abiotic stresses highlighted the significant hormonal regulation of *SmPR‐1* genes, and transcriptome data from CMV‐infected plants confirmed their responsiveness to biological stress, underscoring their role in plant defense mechanisms. These findings provide valuable insights into the molecular functions of *SmPR‐1* genes in stress responses and disease resistance. This study establishes a foundation for future studies exploring the roles of *PR‐1* genes in plant immunity and the development of *S. miltiorrhiza* cultivars with enhanced disease resistance. Further experimental validation and exploration of *PR‐1* gene interactions with other defense pathways are crucial to fully understanding their contributions to plant defense mechanisms.

## Ethics Statement

The authors have nothing to report.

## Conflicts of Interest

The authors declare no conflicts of interest.

## Supporting information


**Table S1.** Protein sequences of all SmPR‐1s.

**Table S2.** Primers used in this study.

## Data Availability

Data will be made available on request.
